# Underestimated Ischemic Heart Disease in Major Adverse Cardiovascular Events after Septicemia Discharge

**DOI:** 10.3390/medicina58060753

**Published:** 2022-05-31

**Authors:** Chih-Chun Hsiao, Yao-Ming Huang, Yin-Han Chang, Hui-Chen Lin, Wu-Chien Chien, Chun-Gu Cheng, Chun-An Cheng

**Affiliations:** 1 Department of Nursing, Taoyuan Armed Forces General Hospital, Taoyuan 32549, Taiwan; hsiaogolden@gmail.com; 2 Department of Emergency Medicine, Taoyuan Armed Forces General Hospital, Taoyuan 32549, Taiwan; algernon8149@gmail.com; 3 Department of Psychology, National Taiwan University, Taipei 10621, Taiwan; caalice2003@yahoo.com.tw; 4 School of Nursing, College of Nursing, Taipei Medical University, Taipei 11031, Taiwan; ceciliatsgh@gmail.com; 5 Graduate Institute of Life Sciences, National Defense Medical Center, Taipei 11490, Taiwan; chienwu@ndmctsgh.edu.tw; 6 Department of Medical Research, Tri-Service General Hospital, National Defense Medical Center, Taipei 11490, Taiwan; 7 School of Public Health, National Defense Medical Center, Taipei 11490, Taiwan; 8 Department of Emergency Medicine, Tri-Service General Hospital, National Defense Medical Center, Taipei 11490, Taiwan; 9 Emergency Department, Department of Emergency and Critical Medicine, Wan Fang Hospital, Taipei Medical University, Taipei 11696, Taiwan; 10 Department of Emergency, School of Medicine, College of Medicine, Taipei Medical University, Taipei 11031, Taiwan; 11 Department of Neurology, Tri-Service General Hospital, National Defense Medical Center, Taipei 11490, Taiwan

**Keywords:** risk, ischemic heart disease, septicemia survivors

## Abstract

*Background and Objectives*: Sepsis increases cardiovascular disease and causes death. Ischemic heart disease (IHD) without acute myocardial infarction has been discussed less, and the relationship between risk factors and IHD in septicemia survivors within six months is worthy of in-depth study. Our study demonstrated the incidence of IHD and the possible risk factors for IHD in septicemia patients within six months. *Materials and Methods*: An inpatient dataset of the Taiwanese Longitudinal Health Insurance Database between 2001 and 2003 was used. The events were defined as rehospitalization of stroke and IHD after discharge or death within six months after the first septicemia hospitalization. The relative factors of major adverse cardiovascular events (MACEs) and IHD were identified by multivariate Cox proportional regression. *Results*: There were 4323 septicemia survivors and 404 (9.3%) IHD. New-onset atrial fibrillation had a hazard ratio (HR) of 1.705 (95% confidence interval (C.I.): 1.156–2.516) for MACEs and carried a 184% risk with HR 2.836 (95% C.I.: 1.725–4.665) for IHD by adjusted area and other risk factors. *Conclusions*: This study explored advanced-aged patients who experienced more severe septicemia with new-onset atrial fibrillation, which increases the incidence of IHD in MACEs within six months of septicemia. Therefore, healthcare providers must identify patients with a higher IHD risk and modify risk factors beforehand.

## 1. Introduction

Sepsis can induce short-term stroke and myocardial infarction within 1 month after discharge [1]. Bacteremia increases the risk of myocardial infarction and ischemic stroke (IS) within 6 months, but insignificantly after 6 months [2]. A past study found that septicemia significantly increased the risk of stroke over 2 years, with a higher risk over 6 months [3]. Long-term major adverse cardiovascular events (MACEs) are affected by sepsis in Taiwan [4].

Sepsis-induced nitric oxide overproduction disrupted calcium influx, which shortened the atrial action potential duration with tachyarrhythmia [5]. The incidence of IS, congestive heart failure (CHF), and death was higher in older patients with sepsis who had received atrial fibrillation (AF) within five years in the United States [6]. Hemodynamic instability and new-onset AF (NOAF) may induce IS and myocardial infarction [7]. A meta-analysis showed that NOAF in sepsis admissions was not related to IS readmission within 6 months [8]. However, a previous study used one-year inpatient data that showed a significant 1.74-fold risk of IS in septicemia survivors within 3 months of NOAF [9].

Past studies have focused on sepsis risk in myocardial infarction [1,4]. It is expected that sepsis is related to myocardial dysfunction and less acute myocardial infarction [10]. Additionally, the incidence of acute coronary syndrome is increased in patients with sepsis compared with non-septic patients [11]. Although coronary artery flow is preserved during sepsis [12], microvascular dysfunction, associated with sepsis through inflammation, immunity, and neuroendocrine function, may induce ischemic heart disease (IHD) [13]. In a previous endotoxin shock study in dogs, the dysfunction of blood flow distribution and oxygen supply mismatching to demand related to focal ischemia offset the oxygen uptake rise in overperfused areas with contractile inefficiency [14]. A past study focused on CHF that possibly progressed from IHD, rather than IHD, in older patients with sepsis [6]. The numbers (7951 in 93,862 patients with sepsis) of CHF were fewer than myocardial infarction (1820), with a higher risk (hazard ratio [HR] 1.48 > 1.22) after sepsis in a past long-term study [4], and some factors seem to be ignored. Past studies have discussed the risk of IHD with coronary artery flow preservation less in the clinical setting. IHD caused by sepsis may induce higher CHF incidence, and this situation seems to be underestimated. We tried to survey this condition.

Most patients with cardiovascular disease have atherosclerosis factors [15], but some are lacking. The number of survivors after sepsis increased after doctors received training and followed sepsis treatment guidelines [16]. We identified the factors associated with MACEs, especially IHD, in septicemia survivors during the higher-risk period within 6 months among the Asian population, according to a previous study [3]. The subsequent IHD and the associated factors for septicemia survivors were worthy of study by a claim dataset from Taiwan.

This study focused on IHD incidence and identified the possible factors and IHD of MACEs in septicemia survivors of an Asian population over a period of six months. We tried to determine the relationship between NOAF and IHD within 6 months after septicemia. For septic patients, physicians must carefully notice modifiable factors to prevent IHD occurrence.

## 2. Materials and Methods

Taiwanese National Health Insurance (NHI) is a unique insurance system developed by the Department of Taiwanese Center National Health Insurance. It was implemented in 1995 for reduced citizens’ payment to search for healthcare support with more than 99% coverage. The National Health Insurance Research Dataset (NHIRD) contains all payment data of healthcare insurance in Taiwan. The dataset included each patient’s date of birth, admission date, sex, disease codes, and operation codes [17].

The inpatient dataset from January 2001 to December 2003 was surveyed. The first-ever septicemia admission was checked by the International Classification of Disease, Ninth Revision, Clinical Modification (ICD-9-CM) codes 038, 003.1, and 036.1, and sepsis was verified [3,4,9]. The new-onset AF was retrieved by ICD-9-CM code 427.3x from the discharge claim data.

**Figure 1:**
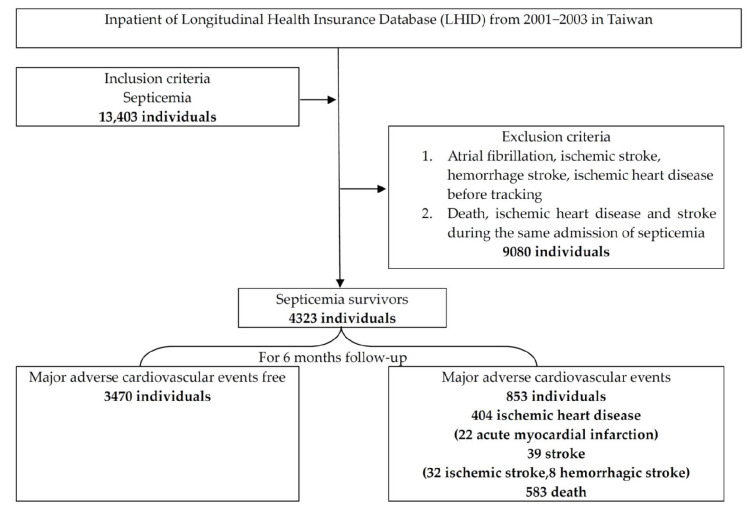
The flowchart of this study.

We defined MACEs with stroke as IS with an ICD-9-CM of 433–437, hemorrhagic stroke with an ICD-9-CM of 431–432, and IHD with an ICD-9-CM of 410–414. Death records were retrieved from the national death database within 6 months. The exclusion criteria were preexisting stroke (ICD-9-CM of 431–437), preexisting IHD, and preexisting AF before first septicemia; the order of sepsis and events of stroke, IHD during the first hospitalization for septicemia and death were not distinguished. Because the NHIRD dataset was recorded during discharge for payments, the order of sepsis and stroke or IHD during hospitalization was not available. We defined acute myocardial infarction (ICD-9 CM 410–411 or ICD-9-OP codes 36.0 and 36.1). The age groups were set for aged effect with youth (aged between 18 and 45 years old), middle-aged (aged between 45 and 65 years old), and senior (aged >65 years old). The ICD-9-CM codes were used to identify comorbid diseases and organ failure according to our previous study [9]. The flowchart of the study is displayed in Figure 1. This study was approved by TSGHIRB B-105-11 (approved on 25 July 2016) by the Tri-Service General Hospital Ethics Institutional Review Board.

Descriptive statistics were performed using the chi-square test (χ^2^) for categorical factors and Student’s *t* test for continuous variables. The cumulative incidences of IHD between the NOAF group and NOAF-free group were analyzed by the log-rank test of Kaplan–Meier curves. The HR of MACEs and IHD for risk factors was determined by multivariate Cox hazard regression. To evaluate the relative factors in NOAF, we used multivariate logistic regression with forward stepwise selection. *p* < 0.05 was defined as statistically significant. The statistical analyses in this study used SPSS software version 21 (Asia Analytics Taiwan Ltd., Taipei, Taiwan).

## 3. Results

**Figure 2:**
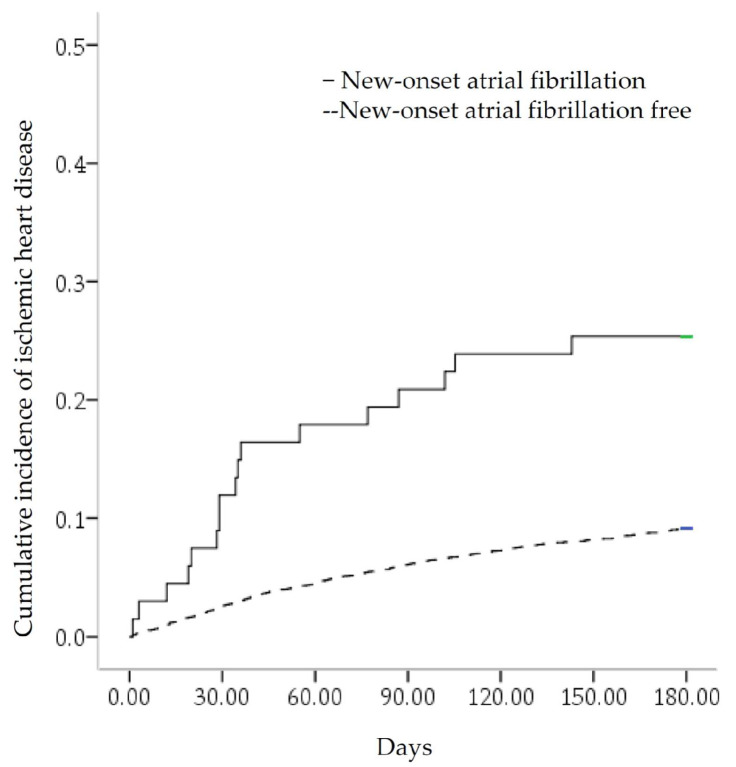
Kaplan–Meier curve for the cumulative incidence of ischemic heart disease. It was stratified by new-onset atrial fibrillation with a log-rank test of *p* < 0.001.

In the study of 4323 septicemia survivors, 853 (19.73%) had MACEs, 39 (0.9%) had stroke, 404 (9.3%) had IHD, and 583 (13.5%) died (Figure 1). There was a total of 41.79% (28/67) MACE occurrence in the NOAF group, compared with 19.38% (825/4256) MACE occurrence in the NOAF-free control group. A total of 25.37% (17/67) of IHDs occurred in the NOAF group, compared with 9.09% (387/4256) of IHD occurrences in the NOAF-free control group (Figure 2). The MACEs in septicemia survivors were older (mean age of 67.6 ± 15.05 years) than those in the MACE-free group (mean age of 58.19 ± 18.89 years), with higher proportions of NOAF, pneumonia, cancer, chronic kidney disease (CKD), chronic obstructive pulmonary disease (COPD), CHF, respiratory, and circulatory, renal and hepatic failure. Males were also predominant, with less hypertension, hyperlipidemia, urinary tract infections, and skin and gastrointestinal infections in the MACE group (Table 1).

The risk factors for MACEs in septicemia survivors were advanced age, with an HR of 1.03 (95% confidence interval [C.I.]: 1.025–1.035]); male sex (HR 1.334 [95% C.I.: 1.153–1.543]); NOAF (HR 1.705 [95% C.I.: 1.156–2.516]); cancer (HR 3.578 [95% C.I.: 3.014–4.246]); CHF (HR 1.52 [95% C.I.: 1.073–2.154]); COPD (HR 1.297 [95% C.I.:1.094–1.538]); CKD (HR 1.53 [95% C.I.: 1.298–1.804]); respiratory failure (HR 1.852 [95% C.I.: 1.549–2.215]); renal failure (HR 1.806 [95% C.I. 1.434–2.274]); and hepatic failure (HR 1.593 [95% C.I.: 1.091–2.328], which adjusted for area and other risk factors (Table 2).

Multivariate Cox proportional regression showed that the risk factors for IHD were advanced age (HR 1.012 [95% C.I.: 1.005–1.018]), male sex (HR 1.266 [95% C.I.: 1.038–1.545]), NOAF (HR 2.836 [95% C.I.: 1.725–4.665]), cancer (HR 4.361 [95% C.I.: 3.441–5.527]), CHF (HR 1.633 [95% C.I.: 1.253–2.128]), COPD (HR 1.37 [95% C.I.: 1.09–1.723]), and CKD (HR 1.79 [95% C.I.: 1.452–2.207), after stepwise forward selection (Table 3). NOAF carries the risk of acute myocardial infarction (crude HR: 12.218 [95% C.I.: 3.503–42.61]) and carried a lower risk of IHD without evidence of acute myocardial infarction (crude HR: 2.944 [95% C.I.: 1.612–5.376]).

The risk factors for stroke were advanced age (HR 1.056 [95% C.I.: 1.029–1.084]), NOAF (HR 3.877 [95% C.I.: 1.342–11.2]), CHF (HR 3.11 [95% C.I.: 1.081–8.945]), metabolic failure during septicemia hospitalization (HR 13.393 [95% C.I.: 3.116–57.556]), and living in outlying islands (HR: 27.472 [95% C.I.: 3.514–214.909]).

There were 61 cardiac deaths and 215 deaths related to malignancy. The risk factors for all-cause death were advanced age (HR 1.038 [95% C.I.: 1.032–1.044]), male sex (HR 1.573 [95% C.I.: 1.325–1.867]), CHF (HR 1.851 [95% C.I.: 1.249–2.745]), CKD (HR 1.308 [95% C.I.: 1.067–1.602]), respiratory failure during septicemia admission (HR 2.272 [95% C.I.: 1.871–2.759]), renal failure during septicemia hospitalization (HR 1.596 [95% C.I.: 1.21–2.105]), hepatic failure during septicemia hospitalization (HR 1.7 [95% C.I.: 1.101–2.623]), cancer (HR 3.703 [95% C.I.: 3.058–4.484]), and living in central (HR 1.363 [95% C.I.: 1.11–1.674]) and Eastern Taiwan (HR 1.641 [95% C.I.: 1.195–2.253]).

The occurrence of NOAF was related to advanced age (OR: 1.077 [95% CI: 1.053–1.101]), peripheral artery disease with OR 5.811 (95% C.I.: 1.277–26.433), and metabolic failure during septicemia hospitalization with an OR of 7.107 (95% C.I.: 1.487–33.961). IHD was associated with a higher risk of death, with an OR of 5.011 (95% C.I.: 4.004–6.272), than stroke with an OR of 2.89 (95% C.I.: 1.456–5.737). We surveyed IHD patients with and without acute myocardial infarction after septicemia discharge. Acute myocardial infarction had a higher risk of death, with an OR of 5.632 (95% C.I.: 2.393–13.254); IHD without acute myocardial infarction had a lower risk of death, with an OR of 4.977 (95% C.I.: 3.956–6.262). The senior septicemia survivors had a higher proportion of NOAF, MACE, stroke, IHD, and death (Table 4).

## 4. Discussion

Our study found that septicemia survivors of advanced age with multiple comorbidities had IHD of MACEs within 6 months. In addition, septicemia survivors with IHD had a higher mortality risk than those with stroke. The healthcare staff in the critical care unit must be aware of these risk factors to prevent a higher number of lethal conditions.

Oxidative–nitrosative stress may contribute to cardiac dysfunction in sepsis. Sepsis increases oxidative stress by activating the isoform of calcium-independent nitric oxygen synthase (iNOS). iNOS can catalyze the formation of reactive nitrogen species (RNS). During sepsis, iNOS is an important source of RNS, as a consequence of enzyme decoupling. In addition, decoupled iNOS is a source of superoxide anions. NO also reacts with superoxide anions to form peroxynitrite anions (ONOO^−^), which oxidize and nitrosylate various biological targets and cause mitochondrial dysfunction [18]. Sepsis-induced oxygen–nitrogen stress triggers lipid peroxidation chain reactions throughout the cardiovascular system and myocardium. These changes lead to the progression of atherosclerosis and the spread of IHD. Thrombosis formation by accentuated platelet activity was shown in viral upper respiratory tract infections and pneumonia [19,20]. Infection increases fibroblasts and chronic reaction protein-related atherosclerosis [21]. Inflammation causes immune dysregulation, higher coagulation, lower oxygenation, and increased neuroendocrine secretion.

Previous studies have shown that, after pneumonia, older and middle-aged patients had a higher risk of cardiovascular disease [22]. Older sepsis survivors have a higher proportion of long-term stroke, CHF, and death in the United States [6]. In our study, age carried a risk of IHD of 1.2% per year of age. As an aging society has been noted worldwide, there is a high risk of an infection developing and progressing to sepsis in elderly individuals. The outbreak of COVID-19 was noted in March 2020, and it is necessary to increase immune activity through exercise cholinergic anti-inflammation enhancement and vaccination to reduce infection rates.

Oxidative stress and inflammatory status contribute to changes in the extracellular matrix, which lead to atrial remodeling and fibrosis, and then result in atrial fibrillation [23]. AF is related to endotoxin effects and vasopressors for hypotension-inducing hypersympathetic stimulation, with persistent calcium channel stimulation and inflammatory action after sepsis [24,25]. A past study in the emergency department found MACEs after infection associated with AF, cardiovascular risk factors, and organ failure [26]. AF in older sepsis patients in America causes 5-year events, which means poor health conditions with more morbidity and mortality [6]. Previous research revealed that 7% of older sepsis patients and 5.8% of septic patients had AF, and this rate was higher in surgical patients [6,23]. The atherosclerosis mechanisms in NOAF of septicemia were cytokine inflammation reactions and higher sympathetic activity with hypercoagulopathy [27]. The occurrence of NOAF is related to advanced age and more comorbid conditions, similar to a previous study [9]. Healthcare providers need to rapidly control the hemodynamic status following sepsis treatment, which could reduce NOAF occurrence and MACE. Our study found that patients who lived in central and eastern parts of Taiwan had MACEs. The potential reason for this was the more severe air pollution in central Taiwan, and fewer hospitals and healthcare providers with heavy workloads who did not follow sepsis guidelines closely in the eastern region. It is necessary to reduce air pollution by government policies and citizens’ cooperation, and educate the physicians in charge of intensive care in these areas of the risks and sepsis guidelines to increase the success of sepsis patient care. One previous study found that NOAF was not a factor in short-term inpatient mortality [28]. Our study investigated similar results within 6 months by providing the necessary treatment for AF after determination, but NOAF could induce subsequent IHD or stroke, which showed reduced life expectancy with indirect effects.

The older males had atherosclerosis factors associated with cardiovascular disease [29]. Our study showed that male patients carried a higher risk. CHF was related to IS in septicemia survivors (OR: 1.39 [95% CI: 1.18–1.63]) in a past study [9]. Our study found a 1.5-fold increased risk of MACEs in septicemia survivors with CHF. There was a 3-fold higher risk of stroke and a 2-fold higher risk of mortality in septicemia survivors with CHF, with only a mildly increased risk of 63.3% in IHD. CHF carries a higher risk of stroke and death, but IHD may progress to CHF. Renal disease is a risk factor for mortality and cardiovascular disease [30]; we found a similar result with a 1.5-fold risk of MACEs and 1.79-fold risk of IHD in septicemia survivors with CKD. Patients with COPD had a 2.0-fold increase in carotid intimal thickness, which is the marker of atherosclerosis [31], and a 30% increased risk of stroke compared with non-COPD patients [32]. The patients with acute exacerbations of COPD had an HR of 3.8 for cardiovascular events in the first 30 days [33]. Our study found a 1.3-fold increased risk in MACEs or IHD. Cancer could induce atherosclerosis by cancer-induced or treatment complications [34]. Our study showed the highest risk of IHD with cancer. Respiratory failure, hepatic failure, renal failure during septicemia hospitalization and tumors decrease life expectancy, which increases the risk of MACEs. However, a lower risk of MACEs was associated with diabetes mellitus and hyperlipidemia in the septicemia survivors included in our study; the intention is for them to receive adequate treatment with diagnostic codes that reduce the risk of MACEs.

The Taiwanese NHIRD covers healthcare services of the majority of the general population, which means that it is generalizable to the population of septicemia survivors. Aspirin prescription can reduce the mortality of community-acquired pneumonia in elderly patients within thirty days, as well as myocardial infarction and IS during hospitalization [35]. Recent studies have shown a poor effect of anticoagulant and beta-blocker use for NOAF after sepsis in lower-level medical care units [36,37], but antiplatelet therapy can reduce IS. In this study, more IHD was found after septicemia discharge, and antiplatelet therapy could be prescribed for primary prevention.

There were some limitations in this study. First, the data were older, and more recent data need to be confirmed in future studies. Second, there is a lack of information on body mass index or some cardiovascular risk factors, such as history and amount of smoking or alcohol consumption, the care course of sepsis, and the vital sign status in the NHIRD by only ICD-9CM codes, which results in incomplete etiology requiring clinical data for in-depth study. Third, elderly septicemia survivors have a higher mortality rate (18.63%), which causes an underestimation of the risk of IHD or stroke in elderly septicemia survivors. Middle-aged sepsis patients with an increased incidence of cardiovascular disease in recent years may have more comorbid conditions due to dietary changes with higher oil and salt consumption that need to be surveyed for possible causes. Fourth, septicemia with CHF was without severity confirmation because of the unavailable patient’s condition and echocardiography report in the claim dataset. Perspective data on the severity of CHF will be used to evaluate the relationship between the degree of CHF and the occurrence of IHD in the future. Fifth, the concentration of basic biomarkers when imaging the clinical condition at the time of discharge (such as lipid metabolism markers, cardiac markers, and inflammatory markers) was unavailable due to the study design by claim data rather than clinical survey. These biochemical parameters need to be checked for the MACEs of sepsis in future studies.

## 5. Conclusions

Septicemia survivors have approximately ten percent of IHD in the follow-up for a period of 6 months. Advanced age and more severe comorbid conditions lead to a higher risk of IHD within 6 months. IHD without acute myocardial infarction was a common complication that was considered less, and IHD was predominantly associated with a six-month decease after septicemia discharge. To elevate post-septic care, healthcare providers must be aware of not only CHF, but also IHD in septicemia survivors.

## Figures and Tables

**Table 1:** Baseline characteristics of the study subjects.

	MACEs (853)	MACEs Free (3470)	*p*
New-onset atrial fibrillation	28 (3.28%)	39 (1.12%)	<0.001 *
Age	67.6 ± 15.05	58.19 ± 18.89	<0.001 *
Gender (male)	503 (58.97%)	1780 (51.3%)	<0.001 *
Hypertension	276 (32.36%)	1273 (36.69%)	0.019 *
Diabetes mellitus	301 (35.29%)	1185 (34.15%)	0.546
Congestive heart failure	35 (4.1%)	62 (1.79%)	<0.001 *
Hyperlipidemia	30 (3.52%)	213 (6.14%)	0.002 *
Chronic kidney disease	219 (25.67%)	543 (15.65%)	<0.001 *
Chronic obstructive pulmonary disease	222 (26.03%)	771 (22.22%)	0.02 *
Peripheral aretery obstructive disease	4 (0.47%)	18 (0.52%)	1
Cancer	183 (21.45%)	180 (5.19%)	<0.001 *
Infection type			
Pneumonia	173 (20.28%)	468 (13.49%)	<0.001 *
Urinary tract infection	230 (27%)	1065 (30.69%)	0.033 *
Skin infection	34 (3.99%)	234 (6.74%)	0.002 *
GI infection	30 (3.52%)	248 (7.15%)	<0.001 *
Hospital type			0.877
Medical center	303 (35.55%)	1201 (34.61%)	
Regional hospital	340 (39.86%)	1397 (40.26%)	
Local hospital	210 (24.61%)	872 (25.13%)	
Area			0.233
Northern Taiwan	346 (40.56%)	1485 (42.8%)	
Central Taiwan	218 (25.57%)	853 (24.58%)	
SouthernTaiwan	223 (26.14%)	929 (26.77%)	
Eastern Taiwan	64 (7.5%)	193 (5.56%)	
Outlying islands	2 (0.23%)	10 (0.29%)	
Organ failure			
Respiratory failure	196 (22.98%)	339 (9.77%)	<0.001 *
Circulatory failure	82 (9.61%)	242 (6.97%)	0.011 *
Renal failure	89 (10.43%)	186 (5.36%)	<0.001 *
Neurologic failure	13 (1.52%)	50 (1.44%)	0.873
Hepatic failure	29 (3.4%)	68 (1.96%)	0.014 *
Metabolic failure	7 (0.82%)	12 (0.35%)	0.078
Hematologic failure	17 (2%)	68 (1.96%)	0.891

* *p* < 0.05; MACEs: major adverse cardiovascular events.

**Table 2:** Risk factors for major adverse cardiovascular events in septicemia survivors.

Risk Factors	Hazard Ratio(95% Confidence Interval)	*p*
New-onset atrial fibrillation	1.705 (1.156–2.516)	0.007 *
Age	1.03 (1.025–1.035)	<0.001 *
Gender (male)	1.334 (1.153–1.543)	<0.001 *
Hypertension	0.631 (0.541–0.737)	<0.001 *
Diabetes mellitus	1.11 (0.954–1.291)	0.177
Congestive heart failure	1.52 (1.073–2.154)	0.018 *
Hyperlipidemia	0.802 (0.554–1.16)	0.241
Chronic kidney disease	1.53 (1.298–1.804)	<0.001 *
Chronic obstructive pulmonary disease	1.297 (1.094–1.538)	0.003 *
Hospital type		0.304
Medical center	reference	
Regional hospital	0.999 (0.844–1.181)	0.987
Local hospital	1.136 (0.939–1.373)	0.189
Area		0.028 *
Northern Taiwan	reference	
Central Taiwan	1.196 (1–1.431)	0.05 *
Southern Taiwan	1.125 (0.948–1.335)	0.176
Eastern Taiwan	1.537 (1.166–2.027)	0.002 *
Outlying islands	1.635 (0.402–6.643)	0.509
Pneumonia	1.176 (0.978–1.416)	0.085
Urinary tract infection	0.862 (0.732–1.015)	0.075
Skin infection	0.704 (0.496–0.997)	0.048 *
GI infection	0.509 (0.352–0.736)	<0.001 *
Respiratory failure	1.852 (1.549–2.215)	<0.001 *
Circulatory failure	0.948 (0.779–1.29)	0.908
Renal failure	1.806 (1.434–2.274)	<0.001 *
Neurological failure	0.784 (0.45–1.365)	0.39
Cancer	3.578 (3.014–4.246)	<0.001 *
Hepatic failure	1.593 (1.091–2.328)	0.016 *
Metabolic failure	1.649 (0.763–3.565)	0.204
Hematologic failure	1.166 (0.719–1.891)	0.533
Peripheral artery obstructive disease	0.948 (0.354–2.538)	0.915

* *p* < 0.05.

**Table 3:** Risk factors for ischemic heart disease in septicemia survivors.

	Univariate Hazard Ratio	*p*	Multivariate Hazard Ratio	*p*
New-onset atrial fibrillation	3.148 (95% C.I.:2.157–4.593)	<0.001 *	2.836 (95% C.I.: 1.725–4.665)	<0.001 *
Age	1.02 (95% C.I.: 1.014–1.026)	<0.001 *	1.012 (95% C.I.:1.005–1.018)	0.001 *
Sex (male)	1.208 (95% C.I.:0.992–1.471)	0.06	1.266 (95% C.I.:1.038–1.545)	0.02 *
Hypertension	0.885 (95% C.I.:0.801–0.977)	0.015 *		
Diabetes mellitus	0.901 (95% C.I.:0.816–0.996)	0.042 *		
Hyperlipidemia	0.978 (95% C.I.:0.796–1.202)	0.831		
Congestive heart failure	2.119 (95% C.I.:1.661–2.704)	<0.001 *	1.633 (95% C.I.:1.253–2.128)	<0.001 *
Chronic kidney disease	2.013 (95% C.I.:1.647–2.462)	<0.001 *	1.79 (95% C.I.:1.452–2.207)	<0.001 *
Cancer	3.684 (95% C.I.:2.924–4.641)	<0.001 *	4.361 (95% C.I:3.441–5.527)	<0.001 *
Chronic obstructive pulmonary disease	1.589 (95% C.I.:1.296–1.969)	<0.001 *	1.37 (95% C.I.:1.09–1.723)	0.007 *
Hospital level				
Medical center	Reference			
Region hospital	1.044 (95% C.I.:0.831–1.311)	0.711		
Local hospital	1.091 (95% C.I.:0.845–1.407)	0.504		
Area				
Northern Taiwan	Reference			
Central Taiwan	0.996 (95% C.I.:0.778–1.277)	0.978		
SouthernTaiwan	1.017 (95% C.I.: 0.8–1.294)	0.89		
Eastern Taiwan	1.064 (95% C.I.:0.699–1.619)	0.772		
Outlying islands	0.87 (0.122–6.214)	0.89		
Infection type				
Pneumonia	1.096 (95% C.I.:0.84–1.429)	0.499		
Urinary tract infection	0.827 (0.662–1.032)	0.092		
Skin infection	0.655 (95% C.I.:0.403–1.064)	0.088		
GI infection	0.585 (95% C.I.:0.355–0.965)	0.036 *		
Organ failure				
Respiratory failure	1.351 (95% C.I.:1.034–1.764)	0.027 *		
Circulatory failure	1.382 (95% C.I.:0.997–1.915)	0.052		
Renal failure	1.613 (95% C.I.:1.16–2.245)	0.006 *		
Neurologic failure	0.676 (95% C.I.:0.253–1.81)	0.436		
Hepatic failure	1.237 (95% C.I.:0.679–2.251)	0.487		
Metabolic failure	2.572 (95% C.I.:0.961–6.886)	0.06		
Hematologic failure	1.034 (95% C.I.:0.514–2.082)	0.925		
Peripheral artery obstructive disease	2.074 (95% C.I.:0.775–5.554)	0.146		

* *p* < 0.05.

**Table 4:** Major adverse cardiovascular events and new-onset atrial fibrillation by age group.

	Youth (18–44 Years Old) (996)	Middle-Aged (45–64 Years Old) (1282)	Senior (>65 Years Old) (2045)
Major adverse cardiovascular disease	77 (7.73%)	245 (19.11%)	531 (25.97%)
Stroke	1 (0.1%)	5 (0.39%)	33 (1.61%)
Ischemic heart disease	43 (4.3%)	126 (9.83%)	235 (11.49%)
Death	44 (4.42%)	158 (12.32%)	381 (18.63%)
New-onset atrial fibrillation	1 (0.1%)	7 (0.55%)	59 (2.89%)

## Data Availability

The datasets used in the current study are available from the corresponding author on reasonable request.

## References

[1] Lai C.-C., Lee M.-T.G., Lee W.-C., Chao C.C.-T., Hsu T.-C., Lee S.-H., Lee C.-C. (2018). Susceptible period for cardiovascular complications in patients recovering from sepsis. CMAJ.

[2] Dalager-Pedersen M., Søgaard M., Schønheyder H.C.C., Nielsen H., Thomsen R.W. (2014). Risk for myocardial infarction and stroke after community-acquired bacteremia: A 20-year population-based cohort study. Circulation.

[3] Lee J.-T., Chung W.-T., Lin J.-D., Peng G.-S., Muo C.-H., Lin C.-C., Wen C.-P., Wang I.-K., Tseng C.-H., Kao C.-H. (2014). Increased risk of stroke after septicaemia: A population-based longitudinal study in Taiwan. PLoS ONE.

[4] Ou S.-M., Chu H., Chao P.-W., Lee Y.-J., Kuo S.-C., Chen T.-J., Tseng C.-M., Shih C.-J., Chen Y.-T. (2016). Long-term Mortality and Major Adverse Cardiovascular Events in Sepsis Survivors: A Nationwide Population-based Study. Am. J. Respir. Crit. Care Med..

[5] Aoki Y., Hatakeyama N., Yamamoto S., Kinoshita H., Matsuda N., Hattori Y., Yamazaki M. (2012). Role of ion channels in sepsis-induced atrial tachyarrhythmias in guinea pigs. Br. J. Pharmacol..

[6] Walkey A.J., Hammill B.G., Curtis L.H., Benjamin E.J. (2014). Long-term outcomes following development of new-onset atrial fibrillation during sepsis. Chest.

[7] Walkey A.J. (2014). Preventing cardiovascular complications of acute infection: A missed opportunity?. Circulation.

[8] Walkey A.J., Wiener R.S., Ghobrial J.M., Curtis L.H., Benjamin E.J. (2011). Incident stroke and mortality associated with new-onset atrial fibrillation in patients hospitalized with severe sepsis. JAMA.

[9] Cheng C.-A., Cheng C.-G., Lin H.-C., Lee J.-T., Lin H.-C., Cheng C.-C., Chien W.-C., Chiu H.-W. (2017). New-Onset Atrial Fibrillation Related Ischemic Stroke Occurring after Hospital Discharge in Septicemia Survivors. QJM.

[10] Zochios V., Valchanov K. (2015). Raised cardiac troponin in intensive care patients with sepsis, in the absence of angiographically documented coronary artery disease: A systematic review. J. Intensive Care Soc..

[11] Wang H.E., Moore J.X., Donnelly J.P., Levitan E.B., Safford M.M. (2017). Risk of Acute Coronary Heart Disease After Sepsis Hospitalization in the REasons for Geographic and Racial Differences in Stroke (REGARDS) Cohort. Clin. Infect. Dis..

[12] Cunnion R.E., Schaer G.L., Parker M.M., Natanson C., Parrillo J.E. (1986). The coronary circulation in human septic shock. Circulation.

[13] Greer J. (2015). Pathophysiology of cardiovascular dysfunction in sepsis. BJA Educ..

[14] Groeneveld A.J., van Lambalgen A.A., van den Bos G.C., Bronsveld W., Nauta J.J., Thijs L.G. (1991). Maldistribution of heterogeneous coronary blood flow during canine endotoxin shock. Cardiovasc. Res..

[15] O’Donnell M.J., Xavier D., Liu L., Zhang H., Chin S.L., Rao-Melacini P., Rangarajan S., Islam S., Pais P., McQueen M.J. (2010). Risk factors for ischaemic and intracerebral haemorrhagic stroke in 22 countries (the INTERSTROKE study): A case-control study. Lancet.

[16] Iwashyna T.J., Cooke C.R., Wunsch H., Kahn J.M. (2012). Population Burden of Long-Term Survivorship After Severe Sepsis in Older Americans. J. Am. Geriatr. Soc..

[17] (2022). National Health Insurance Research Database Taipei. http://nhird.nhri.org.tw/en/index.html.

[18] Neri M., Riezzo I., Pomara C., Schiavone S., Turillazzi E. (2016). Oxidative-nitrosative stress and myocardial dysfunctions in sepsis: Evidence from the literature and postmortem observations. Mediat. Inflamm..

[19] Kreutz R.P., Tantry U.S., Bliden K.P., Gurbel P.A. (2007). Inflammatory changes during the ‘common cold’are associated with platelet activation and increased reactivity of platelets to agonists. Blood Coagul. Fibrinolysis.

[20] Santos-Gallego C.G., Badimon J.J. (2014). The sum of two evils: Pneumonia and myocardial infarction: Is platelet activation the missing link?. J. Am. Coll. Cardiol..

[21] Emsley H.C., Hopkins S.J. (2008). Acute ischaemic stroke and infection: Recent and emerging concepts. Lancet Neurol..

[22] Corrales-Medina V.F., Alvarez K.N., Weissfeld L.A., Angus D.C., Chirinos J.A., Chang C.-C.H., Newman A., Loehr L., Folsom A.R., Elkind M.S. (2015). Association between hospitalization for pneumonia and subsequent risk of cardiovascular disease. JAMA.

[23] Schwartz A., Brotfain E., Koyfman L., Klein M. (2015). Cardiac Arrhythmias in a Septic ICU Population: A Review. J. Crit. Care Med..

[24] Werdan K., Schmidt H., Ebelt H., Zorn-Pauly K., Koidl B., Hoke R.S., Heinroth K., Müller-Werdan U. (2009). Impaired regulation of cardiac function in sepsis, SIRS, and MODS. Can. J. Physiol. Pharmacol..

[25] De Groot B., van den Berg S., Kessler J., Ansems A., Rijpsma D. (2016). Independent predictors of major adverse cardiovascular events in emergency department patients who are hospitalised with a suspected infection: A retrospective cohort study. BMJ Open.

[26] Christian S.A., Schorr C., Ferchau L., Jarbrink M.E., Parrillo J.E., Gerber D.R. (2008). Clinical characteristics and outcomes of septic patients with new-onset atrial fibrillation. J. Crit. Care.

[27] Kuipers S., Klouwenberg P.M.K., Cremer O.L. (2014). Incidence, risk factors and outcomes of new-onset atrial fibrillation in patients with sepsis: A systematic review. Crit. Care.

[28] Carrera P., Thongprayoon C., Cheungpasitporn W., Iyer V.N., Moua T. (2016). Epidemiology and outcome of new-onset atrial fibrillation in the medical intensive care unit. J. Crit. Care.

[29] Berry J.D., Dyer A., Cai X., Garside D.B., Ning H., Thomas A., Greenland P., Van Horn L., Tracy R.P., Lloyd-Jones D.M. (2012). Lifetime risks of cardiovascular disease. N. Engl. J. Med..

[30] Weiner D.E., Tighiouart H., Amin M.G., Stark P.C., MacLeod B., Griffith J.L., Salem D.N., Levey A.S., Sarnak M.J. (2004). Chronic kidney disease as a risk factor for cardiovascular disease and all-cause mortality: A pooled analysis of community-based studies. J. Am. Soc. Nephrol..

[31] Sin D.D., MacNee W. (2013). Chronic obstructive pulmonary disease and cardiovascular diseases: A “vulnerable” relationship. Am. J. Respir. Crit. Care Med..

[32] Kim Y.R., Hwang I.C., Lee Y.J., Ham E.B., Park D.K., Kim S. (2018). Stroke risk among patients with chronic obstructive pulmonary disease: A systematic review and meta-analysis. Clinics.

[33] Kunisaki K.M., Dransfield M.T., Anderson J.A., Brook R.D., Calverley P.M.A., Celli B.R., Crim C., Hartley B.F., Martinez F.J., Newby D.E. (2018). Exacerbations of Chronic Obstructive Pulmonary Disease and Cardiac Events. Am. J. Respir. Crit. Care Med..

[34] Roubín S.R., Cordero A. (2019). The Two-way Relationship Between Cancer and Atherosclerosis. Rev. Esp. Cardiol..

[35] Falcone M., Russo A., Cangemi R., Farcomeni A., Calvieri C., Barillà F., Scarpellini M.G., Bertazzoni G., Palange P., Taliani G. (2015). Lower Mortality Rate in Elderly Patients With Community-Onset Pneumonia on Treatment With Aspirin. J. Am. Heart Assoc..

[36] Walkey A.J., Quinn E.K., Winter M.R., McManus D.D., Benjamin E.J. (2016). Practice Patterns and Outcomes Associated With Use of Anticoagulation Among Patients with Atrial Fibrillation During Sepsis. JAMA Cardiol..

[37] Walkey A.J., Evans S.R., Winter M.R., Benjamin E.J. (2016). Practice patterns and outcomes of treatments for atrial fibrillation during sepsis: A propensity-matched cohort study. Chest.

